# Staff perceptions of caring for people exhibiting behavioural and psychological symptoms of dementia in residential aged care: A cross‐sectional survey

**DOI:** 10.1111/ajag.12734

**Published:** 2019-10-15

**Authors:** Jessica Roe, Suzanne Coulson, Cherene Ockerby, Alison M. Hutchinson

**Affiliations:** ^1^ School of Nursing and Midwifery Deakin University Geelong Vic. Australia; ^2^ Centre for Quality and Patient Safety Research Monash Health Melbourne Vic. Australia

**Keywords:** behavioural symptoms, caregivers, dementia, nursing staff, residential facilities

## Abstract

**Objectives:**

To explore the attitudes of direct care staff in residential aged care when interacting with, and responding to, residents exhibiting behavioural and psychological symptoms of dementia (BPSD).

**Methods:**

Cross‐sectional survey (n = 70).

**Results:**

Participants favoured a person‐centred approach to their management of residents with BPSD and were aware of the causes of BPSD. There were significant differences between personal care workers’ (PCWs) and enrolled nurses’ (ENs) perceptions of the impact of interpersonal relationships and the physical environment on aggressive behaviours, and between registered nurses and both PCWs and ENs regarding the use of medications for aggressive behaviour and a medical approach to care.

**Conclusions:**

The attitudes of participants reflected an awareness of BPSD and its causes. Participants recognised the benefits of a person‐centred paradigm, but more education directed towards ENs and PCWs regarding appropriate administration and potential risks of psychotropic medication for BPSD may be beneficial.

**Policy Impact**

Understanding the attitudes of staff in relation to caring for people with behavioural and psychological symptoms of dementia can help guide policymakers, leaders and managers in decision‐making about resource allocation, and training and education needs.

**Practice Impact**

All levels of direct care staff favoured person‐centred care in the management of behavioural and psychological symptoms of dementia. Further education may be required for some staff groups to raise awareness of potential risks of psychotropic medication and ensure they have the knowledge and skills to provide evidence‐based care and appropriate medication administration.

## 1 INTRODUCTION

Behavioural and psychological symptoms of dementia (BPSD) are common, particularly among persons living in residential aged care facilities (RACFs). Over half of the people living in RACFs in Australia have a diagnosis of dementia^1^ and at least 80% of these people experience BPSD.^2^ The prevalence of dementia is expected to reach 900 000 in Australia by 2050, almost triple that of the current prevalence.^1^ Determining the most effective and appropriate strategies for direct care staff to use when caring for people with BPSD is of paramount importance to ensure these people are safe, experience the best quality of life and continue to have positive interactions with family, friends and staff. Use of such strategies is also important for achieving a sustainable and healthy workforce that is positioned to provide optimal care for people with dementia in Australia's RACFs. An understanding of the attitudes of RACF direct care staff in relation to management of BPSD can guide policymakers, leaders and managers in decision‐making about resource allocation, and training and education needs.

### 1.1 Background

Within the context of Australia's ageing population, and the expected dramatic increase in the prevalence of dementia, unprecedented demands will be placed on RACFs. Currently, 60% of people with dementia are placed permanently in an RACF at some stage during the process of their disease.^3^ Providing adequate care for these people remains a challenge in a sector that struggles to attract and retain adequate and sustainable fiscal resources.^4^ Further complicating this situation is the challenge of retaining direct care staff and, in particular, registered nurses (RNs), as evidenced by significantly higher staff turnover rates compared to those of other health‐care sectors in Australia.^5^

One particular challenge for direct care staff of RACFs is providing quality care to residents experiencing BPSD. Common examples of symptoms include physical and verbal aggression, agitation, wandering, sleep disturbances, depression and anxiety, and inappropriate sexual behaviour. Caring for people with BPSD is challenging for direct care staff who are striving to meet the needs of a number of residents. This challenge has been found to negatively affect the physical and psychological health of staff and contribute to sick leave, absenteeism and staff turnover.^6^, ^7^, ^8^, ^9^ Additionally, it has been found that staffing issues, such as sick leave, absenteeism and turnover, related to the incidence of BPSD, result in a vicious circle, in some instances increasing the occurrence of BPSD due to poor staff‐to‐resident ratios and staff fatigue. Consequently, the presence of BPSD results in additional costs for the RACF industry and heavy demands on staff, which impacts directly on the facilities, and their staff's ability to provide quality care to residents. Subsequently, staff may fail to meet the needs of these residents.^10^

Current guidelines recommend person‐centred interventions as first‐line treatment for BPSD.^2^ These interventions include environmental changes (for comfort, or to meet personal preferences of the person with BPSD), finding interesting and enjoyable activities suitable for the individual, and recognising and responding to unmet needs. Due to the severe physical and psychological risks (including stroke and death) associated with the use of psychotropic medication for BPSD,^11^ the Australian National Health and Medical Research Council guidelines recommend that these medications be used only if all other strategies fail.^2^ Despite these recommendations, the routine administration of psychotropic medication, specifically antipsychotics or antianxiety agents, for the treatment of BPSD is common practice in Australia and other Western countries, with up to 78% of residents in RACFs having been prescribed some form of psychotropic medication.^12^

There is a clear discrepancy between the best practice recommendations for managing BPSD in RACFs and how BPSD is actually managed in this context. Previous research has demonstrated that although direct care staff in RACFs in Australia most commonly favour person‐centred approaches to managing BPSD, psychotropic medication for BPSD was administered more often than not, due to time and resource constraints, and the complexities involved in implementing person‐centred interventions.^13^, ^14^ Lack of training or education, as well as organisational structures and the inherent culture, have been identified as barriers to implementing person‐centred interventions for BPSD.^14^

### 1.2 Objectives

The aim of this research was to gain an understanding of the attitudes of direct care staff in an RACF when interacting with residents displaying BPSD. Specific objectives were to:

1. Identify staff knowledge and attitudes towards residents experiencing BPSD.
2. Determine whether there was a relationship between attitudes towards BPSD and role designation.
3. Determine whether there was a relationship between role designation and perceptions of providing person‐centred care to persons with BPSD.

## 2 METHODS

### 2.1 Design

A cross‐sectional survey was administered.

### 2.2 Research setting and participants

This research was undertaken in one health‐care organisation that provided both community‐based and residential aged care services in regional Victoria, Australia. The organisation operated three residential departments, which were home to approximately 200 residents, of whom 110 (55%) had a diagnosis of moderate‐to‐severe dementia. Of the 410 staff employed by the organisation, this study targeted the 173 direct care staff working across the residential departments. The residential departments were selected because staff working in these settings had more exposure to BPSD than staff in the other community‐based departments within the organisation. For the purposes of this research, the direct care staff included registered nurses (RNs), enrolled nurses (ENs) and personal care workers (PCWs) who were employed in clinical roles at the time of data collection.

### 2.3 Data collection instrument

The “Management of Aggression in People with Dementia Attitude Questionnaire” (MAPDAQ)^15^ was used to survey the staff. This 20‐item instrument was adapted specifically for use in RACFs from the “Management of Aggression and Violence Attitude Scale” (MAVAS).^16^ Participants rate the extent of their agreement with each of the items on a visual analogue scale. Responses to each item reflect a numeric score, ranging from 0 to 100, with lower scores indicating greater agreement with the statement (strongly agree = 0, strongly disagree = 100). Pulsford et al^15^ reported a test‐retest reliability estimate, using Pearson's *r* coefficient, of .817 which is indicative of good reliability.^17^ They also undertook a factor analysis, which resulted in two factors accounting for 19 items.^15^ The factors were interpreted to represent the “standard paradigm perspective” (which reflects the traditional biomedical model of health‐care provision) and the more contemporary “person‐centred perspective.” These factors were adopted as a framework for reporting the results of the current research. Note that the item that did not load on either factor (Item 9) was retained in the current study but excluded from analyses based on the two factors. Basic demographic data were also collected including sex, age group, years worked at the facility, years of practice and education level.

### 2.4 Data collection

A census sampling approach was adopted, with all permanently employed staff providing care for residents living in the residential units invited to complete the questionnaire. This included 27 RNs, 76 ENs and 70 PCWs. The questionnaire was distributed by an independent representative and contact person for this research, nominated by senior management within the organisation. Surveys were distributed in September 2016.

To maximise the response rate, potential participants were informed of the questionnaire and the broad aims of the research via email, posters and information sessions during handover. The MAPDAQ was distributed by the independent representative in hard copy to all staff in an envelope that was personally addressed and included a plain language statement, questionnaire and an envelope in which to return the survey anonymously once completed. Flyers in the nurses’ stations and tea rooms reminded staff to return completed questionnaires to the designated drop‐box.

### 2.5 Data analysis

Data were analysed using IBM SPSS Statistics version 24, including descriptive and inferential statistics. Data were not normally distributed so median (Md) and interquartile range (IQR) are reported. In accordance with the work of Pulsford et al,^15^ MAPDAQ data are reported based on the two factor structure of the tool (ie person‐centred perspective and standard paradigm perspective). The questions were further classified into two subcategories, causation (ie causes of aggressive behaviour) and management (ie responding to aggressive behaviour) within the two domains.

Due to the non‐normal distribution of the data, as well as the modest sample size within some groups, non‐parametric comparative tests were used. Kruskal‐Wallis tests were performed to compare the responses of RNs, ENs and PCWs, with post hoc Mann‐Whitney *U* tests conducted to determine which groups differed significantly from each other. A Bonferroni adjustment was applied to the alpha level of the post hoc tests to control for Type 1 error, with a revised alpha value of 0.017. The effect size (*r*) was calculated by dividing the *z* value by the square root of n.

### 2.6 Ethics approval

Low‐risk ethics approval was granted through the Deakin University Human Ethics Advisory Group for the Faculty of Health (HEAG‐H 112_2016), and this approval was accepted by the aged care organisation in which the research was conducted.

## 3 RESULTS

### 3.1 Participant demographic characteristics

Seventy surveys were completed (40% response rate). The demographic characteristics of participants are outlined in Table 1. Just over half of participants were ENs (51%) and, accordingly, 51% of participants had completed either a Certificate IV/Diploma of Nursing or a hospital‐based Nursing Certificate. The majority of respondents were aged between 46 and 65 years, and the median number of years worked at the facility was 10.0.

**Table 1:** Demographic characteristics of survey respondents (n = 70)

Characteristic	n	%
Gender		
Female	54	77
Male	3	4
Missing	13	19
Role		
RN	12	17
Enrolled nurse	36	51
PCW	20	29
Missing	2	3
Age group (y)		
18‐25	1	1
26‐35	7	10
36‐45	14	20
46‐55	24	34
56‐65	20	29
65+	1	1
Missing	3	4
Education		
Secondary School	2	3
Certificate III	17	24
Certificate IV/ Diploma of Nursing	22	31
Nursing Certificate (Hospital Training)	14	20
Bachelor's Degree	7	10
Bachelor's Degree (Honours)	1	1
Postgraduate Certificate	4	6
Postgraduate Diploma	1	1
Master's Degree	0	0
PhD/Doctorate	0	0
Missing	2	3

	Range	Md	IQR
Years at residential aged care facility (n = 61)	1‐33	10.0	10.5
Years practising as Nurse/PCW (n = 41^a^)	2‐50	12.0	16.5
Years practising in Residential Aged Care (n = 42^a^)	1‐40	10.0	14.3

Abbreviations: IQR, interquartile range; Md, median; PCW, personal care worker.

a The sample size for these items is reduced due to a large amount of missing data.

### 3.2 Management of People with Dementia Attitude Questionnaire (MAPDAQ)

Overall, participants favoured the person‐centred paradigm (Factor 1) with responses in this section generally being in agreement with the statements (ie lower scores) (Table 2). Participants strongly agreed with the statement “people with dementia may be aggressive because they are in pain,” and there was generally more agreement with statements about causes of aggression (eg restrictive environment, staff not listening to the resident) in the person‐centred paradigm than in the standard paradigm perspective (Factor 2). The exception was Item 4 in the standard paradigm perspective, “people with dementia are aggressive because of the illness that they have,” with which participants generally agreed. Of the questions relating to the management of aggressive behaviour, participants most strongly agreed with items 14 and 18, suggesting that staff viewed the use of distraction as well as their relationship with the resident as useful management strategies for aggressive behaviour. The results from Table 2 show the participants disagreed with the use of isolation or physical restraint for aggressive behaviour and, while there was a tendency towards agreement that medications were valuable in managing aggression, respondents also agreed that alternatives to medication could be used more frequently in their workplace.

**Table 2:** MAPDAQ results by staff role designation (n = 70)

Statement	Overall (n = 70)		RN (n = 12)		Enrolled nurse (n = 36)		Personal care worker (n = 20)	
	Md	IQR	Md	IQR	Md	IQR	Md	IQR
Factor 1—Person‐centred perspective	25.6	16.6	24.7	15.3	27.2	21.2	22.1	11.0
a) Causes of aggressive behaviour								
2. If staff do not listen to residents with dementia, they may become aggressive	20.3	29.3	17.8	13.8	21.3	32.0	15.3	42.2
20. Residents with dementia may be aggressive because they don't understand what staff are trying to do for them	13.1	22.0	13.1	17.4	15.2	23.7	10.1	18.4
1. Other people make people with dementia aggressive	42.4	50.6	49.5	45.5	38.8	53.0	52.5	71.6
16. People with dementia may be aggressive because they are in pain	5.1	9.9	5.1	3.0	5.1	13.1	7.1	10.1
11. Restrictive environments can contribute towards aggression	17.7	30.3	21.2	25.5	18.2	47.2	15.2	22.7
19. If the physical environment were different, people with dementia would be less aggressive	34.3	37.4	38.4	33.1	43.9	36.6	17.7	36.6
b) Responding to aggressive behaviour: General views								
15. Aggression could be handled more effectively in this Home	37.9	58.6	43.9	67.7	31.8	50.3	34.3	64.9
c) Responding to aggressive behaviour: Use of non‐physical methods								
10. Talking to the person is an effective way of managing aggression	32.8	34.3	43.4	42.4	33.8	29.8	18.2	41.9
14. Improved relationships between staff and residents with dementia can reduce aggression	16.2	30.8	12.1	15.4	20.7	34.6	9.6	15.7
18. The use of distraction is helpful in managing aggression	14.1	20.2	15.2	17.7	17.2	30.1	8.6	18.7
Factor 2—Standard paradigm perspective	60.0	21.3	70.7	18.9	58.5	17.6	58.6	14.0
a) Causes of aggressive behaviour								
3. People with dementia are aggressive because that's their personality	87.9	22.4	91.9	20.2	88.4	19.9	82.1	25.0
4. People with dementia are aggressive because of the illness that they have	26.4	43.6	42.9	49.1	30.0	38.5	15.2	48.0
5. People with dementia should control their feelings	90.8	14.5	91.9	8.3	91.9	16.8	90.4	20.2
b) Responding to aggressive behaviour: Use of medication								
8. Medication is a valuable approach for managing aggression	38.4	43.4	43.4	60.3	34.7	41.8	34.2	33.2
13. Alternatives to medication could be used more frequently in this Home	34.3	41.7	36.9	30.8	31.3	48.2	38.9	44.4
17. Prescribed medication should be used more frequently for aggressive behaviour	39.4	34.3	73.2	56.6	36.4	33.1	38.4	40.4
c) Responding to aggressive behaviour: Use of isolation								
6. Staff should be able to isolate an aggressive resident in a separate room	51.5	63.3	74.1	82.1	46.5	57.4	51.3	67.2
d) Responding to aggressive behaviour: Use of restraint								
7. People with dementia who are aggressive should be physically restrained for their own safety or the safety of others	89.8	32.7	90.7	15.3	89.8	36.1	87.6	39.8
12. Physical restraint is used more than necessary in this Home	96.0	13.9	98.0	7.1	95.5	16.2	93.9	17.2
Additional free‐standing item								
9. People with dementia will calm down if left alone	54.0	53.7	65.0	34.4	56.9	50.8	44.2	69.8

Abbreviations: IQR, interquartile range; MAPDAQ, Management of Aggression in People with Dementia Attitude Questionnaire; Md, median.

A comparison of MAPDAQ factor scores based on staff designation (ie RN, EN and PCW) identified statistically significant differences based on role for Factor 2 (standard paradigm perspective), χ^2^ (2, n = 61) = 7.69, *P *= .021. Post hoc Mann‐Whitney *U* tests revealed that the RNs recorded a higher median score (Md 70.7) indicative of stronger disagreement than both the ENs (Md 58.6; *U* = 81.0, *z* = −2.49, *P *= .013, *r* = .38) and the PCWs (Md 58.6; *U* = 32.0, *z* = −2.66, *P* = .008, *r* = .51). For Factor 1 (person‐centred perspective), there was no significant difference in the responses of staff across the three groups, χ^2^ (2, n = 57) = 2.43, *P* = .297.

Significant differences, based on role, were identified for four of the individual MAPDAQ items. For Item 14 (improved relationships between staff and residents with dementia can reduce aggression; χ^2^ [2, n = 68] = 7.93, *P* = .019), post hoc analysis revealed a higher level of agreement by the PCWs (Md 9.6) compared with the ENs (Md 20.7; *U* = 211.5, *z* = −2.54, *P* = .011, *r* = .34). Similarly, for Item 19 (if the physical environment were different, people with dementia would be less aggressive; χ^2^ [2, n = 68] = 7.31, *P* = .026), the PCWs agreed more strongly (Md 17.7) compared with the ENs (Md 43.9; *U* = 202.0, *z* = −2.70, *P* = .007, *r* = .36). For Item 17 (prescribed medication should be used more frequently for aggressive behaviour; χ^2^ [2, n = 67] = 7.91, *P* = .019), the RNs’ responses showed stronger disagreement (Md 73.2) than both the ENs (Md 36.4; *U* = 103.0, *z* = −2.69, *P* = .007, *r* = .39) and PCWs (Md 38.4; *U* = 59.0, *z* = −2.23, *P* = .026, *r* = .40). Finally, for Item 10 (talking to the person is an effective way of managing aggression; χ^2^ [2, n = 59] = 6.63, *P* = .036) post hoc tests did not reach statistical significance once the Bonferroni adjustment was applied. There was a trend towards higher agreement by the PCWs (Md 18.2) than both the RNs (Md 43.4; *U* = 40.5, *z* = −2.24, *P* = .025, *r* = .43) and the ENs (Md 33.8; *U* = 168.0, *z* = −2.19, *P* = .029, *r* = .31).

## 4 DISCUSSION

The primary aim of this research was to gain an understanding of the attitudes of direct care staff in regard to caring for residents who exhibit BPSD in residential aged care. The results from the MAPDAQ showed that participants, especially the PCW group, felt strongly that the environment is a cause of BPSD in residents. Participants agreed with statements suggesting that causes of BPSD are situational and a result of the immediate environment, rather than an expected symptom of the disease, or a personality trait. This finding is consistent with previous MAPDAQ research, which identified that direct care staff perceived the causes of BPSD to be predominantly situational.^15^ This finding suggests the direct care staff generally understand the underlying triggers for BPSD in residents.

It was clear that all designations of staff strongly favoured person‐centred care, recognising the important role that individualised interventions play in maintaining the health and well‐being of residents with dementia. The survey uncovered staff preferences for a person‐centred paradigm, rather than the traditional medical model of care. This finding was consistent with the findings of a UK study in which the MAPDAQ questionnaire was first used. The researchers concluded that staff recognised and favoured the benefits of, and had a rationale for using, a person‐centred paradigm rather than the traditional paradigm in their management of residents with BPSD.^15^ This is in line with recent worldwide trends, which advocate for the use of a person‐centred approach, focusing on the person first, rather than the traditional task‐orientated biomedical approach to dementia care.^18^ The traditional biomedical approach is now recognised to be reductionistic and inadequate for the care of people living with dementia, as it fails to address factors such as individual perspectives of well‐being and self‐worth.^19^

This research explored and compared the attitudes of RNs, ENs and PCWs about BPSD and its subsequent management. Staff attitudes indicated that they had a good understanding of the causes and management of BPSD with the majority of staff viewing BPSD as symptomatic of an unmet need or a response to an unsuitable environment, which are views in line with current research and evidence‐based clinical guidelines for dementia care.^2^

While all staff typically disagreed with the items in the MAPDAQ standard paradigm perspective, RNs disagreed more strongly with this overall factor than both the ENs and PCWs. This pattern was evident in relation to the administration of medication for BPSD. The survey revealed that while RNs disagreed that medication should be used more frequently for BPSD, ENs and PCWs tended to agree with this statement. This finding highlights a possible lack of understanding by ENs and PCWs about the risks of antipsychotic use in people with dementia. This is particularly important given that these two staff designations make up the bulk of the care staff. This gap in knowledge is understandable given that staff in these roles have typically not had specific training regarding the administration of antipsychotics or medications more generally.

The present research is the first to use the MAPDAQ to compare and contrast attitudes of RNs, ENs and PCWs regarding person‐centred care vs pharmacological management of BPSD in RACFs. The findings indicate there is a need to provide further training to direct care staff, in particular ENs and PCWs, on appropriate use of medications for BPSD. This is an important finding because although ENs and PCWs usually need the authorisation of an RN to administer these medications, due to staffing models in RACFs the RN often relies on the clinical judgement of the direct care staff in making their decisions to administer antipsychotic medications.

### 4.1 Limitations

A limitation of this research was the response rate of only 40%; however, this is still respectable based on research, suggesting the average response rate for health‐care workers in Australia was 53% for paper‐based questionnaires.^20^ Recruitment of staff from a single organisation limits the generalisability of the findings, which may not be representative of all RACFs in Australia. Finally, there is potential for social desirability bias associated with the self‐report method, whereby participants tend to respond in a manner that would be viewed favourably rather than providing an accurate response. To minimise this risk, the survey was distributed by an independent person with no managerial responsibility for participants. Additionally, the anonymity and confidentiality of the survey was stated in the covering letter, emphasised at information sessions and reinforced by the provision of sealable envelopes into which participants placed their completed surveys.

## 5 CONCLUSIONS

Due to demographic trends in health and the ageing population, Australia is facing a substantial increase in people being diagnosed with dementia, which is expected to place unprecedented demands on Australian residential aged care services. This research demonstrated that all levels of direct care staff working in RACF overwhelmingly favoured person‐centred care in the management of BPSD, recognising the importance of providing individualised care to each resident and attempting to provide interventions based on unmet needs and personal preferences. Further education may be required for some staff groups, however, to ensure they have the requisite knowledge and skills to provide evidence‐based person‐centred care with minimal reliance on psychotropic medications.

## CONFLICTS OF INTEREST

No conflicts of interest declared.

## References

[1] Australian Institute of Health and Welfare . Dementia in Australia. Cat. No. AGE 70. Canberra, ACT: AIHW; 2012.

[2] Guideline Adaptation Committee . Clinical Practice Guidelines and Principles of Care for People with Dementia. Sydney, NSW: Guideline Adaptation Committee; 2016.

[3] Australian Institute of Health and Welfare . Austalia's Welfare 2015. Austalia's Welfare Series no. 12 Cat. No. AUS 189. Canberra, ACT: AIHW; 2015.

[4] Ergas H , Paolucci F . Providing and financing aged care in Australia. Risk Manag Healthc Policy. 2011;4:67‐80. 22312229 10.2147/RMHP.S16718 PMC3270928

[5] Baldwin R , Kelly J , Sharp D . The Aged Care Workforce in Australia: White Paper. Canberra, ACT: Aged and Community Services Australia; 2014.

[6] Aström S , Karlsson S , Sandvide A , et al. Staff's experience of and the management of violent incidents in elderly care. Scand J Caring Sci. 2004;18(4):410‐416. 15598249 10.1111/j.1471-6712.2004.00301.x

[7] Black W , Almeida OP . A systematic review of the association between the behavioral and psychological symptoms of dementia and burden of care. Int Psychogeriatr. 2004;16(3):295‐315. 15559754 10.1017/s1041610204000468

[8] Cubit K , Farrell G , Robinson A , Myhill M . A survey of the frequency and impact of behaviours of concern in dementia on residential aged care staff. Australas J Ageing. 2007;26(2):64‐70.

[9] Koder DA . The psychosocial management of challenging behaviours in rehabilitation settings: the importance of nursing staff involvement. J Australas Rehabil Nurse Assoc. 2011;14(3):8‐12.

[10] Battaglini E . Aggression in the Elderly: Risk Factors and Management. Adelaide, SA: The Joanna Briggs Institute; 2014.

[11] Kunde L . Evidence Summary. Aggression in Older People: Antipsychotics (JBI8444). Adelaide, SA: The Joanna Briggs Institute; 2014.

[12] Nishtala PS , McLachlan AJ , Bell JS , Chen TF . Psychotropic prescribing in long‐term care facilities: impact of medication reviews and educational interventions. Am J Geriatr Psychiatry. 2008;16(8):621‐632. 18669940 10.1097/JGP.0b013e31817c6abe

[13] Borbasi S , Emmanuel E , Farrelly B , Ashcroft J . Report of an evaluation of a nurse‐led dementia outreach service for people with the behavioural and psychological symptoms of dementia living in residential aged care facilities. Perspect Public Health. 2011;131(3):124‐130. 21692400 10.1177/1757913911400143

[14] Ervin K , Finlayson S , Cross M . The management of behavioural problems associated with dementia in rural aged care. Collegian. 2012;19(2):85‐95. 22774350 10.1016/j.colegn.2012.02.003

[15] Pulsford D , Duxbury JA , Hadi M . A survey of staff attitudes and responses to people with dementia who are aggressive in residential care settings. J Psychiatr Ment Health Nurs. 2011;18(2):97‐104. 21299721 10.1111/j.1365-2850.2010.01646.x

[16] Duxbury J . Testing a new tool: the Management of Aggression and Violence Attitude Scale (MAVAS). Nurse Res. 2003;10(4):39‐52. 12838818 10.7748/nr2003.07.10.4.39.c5906

[17] Pallant J . SPSS Survival Manual: A Step by Step Guide to Data Analysis using SPSS for Windows (4th ed.). Sydney, NSW: Allen & Unwin; 2011.

[18] Hayajneh FA , Shehadeh A . The impact of adopting person‐centred care approach for people with Alzheimer's on professional caregivers' burden: an interventional study. Int J Nurs Pract. 2014;20(4):438‐445. 25039325 10.1111/ijn.12251

[19] Edvardsson D , Winblad B , Sandman PO . Person‐centred care of people with severe Alzheimer's disease: current status and ways forward. Lancet Neurol. 2008;7(4):362‐367. 18339351 10.1016/S1474-4422(08)70063-2

[20] Cook JV , Dickinson HO , Eccles MP . Response rates in postal surveys of healthcare professionals between 1996 and 2005: an observational study. BMC Health Serv Res. 2009;9:160. 19751504 10.1186/1472-6963-9-160 PMC2758861

